# Alternate Reading Frame Protein (F Protein) of Hepatitis C Virus: Paradoxical Effects of Activation and Apoptosis on Human Dendritic Cells Lead to Stimulation of T Cells

**DOI:** 10.1371/journal.pone.0086567

**Published:** 2014-01-27

**Authors:** Subodh Kumar Samrat, Wen Li, Shakti Singh, Rakesh Kumar, Babita Agrawal

**Affiliations:** 1 Department of Surgery, Faculty of Medicine and Dentistry, University of Alberta, Edmonton, Alberta, Canada; 2 Department of Laboratory Medicine and Pathology, Faculty of Medicine and Dentistry, University of Alberta, Edmonton, Alberta, Canada; INRS - Institut Armand Frappier, Canada

## Abstract

Hepatitis C virus (HCV) leads to chronic infection in the majority of infected individuals due to lack, failure, or inefficiency of generated adaptive immune responses. In a minority of patients, acute infection is followed by viral clearance. The immune correlates of viral clearance are not clear yet but have been extensively investigated, suggesting that multispecific and multifunctional cellular immunity is involved. The generation of cellular immunity is highly dependent upon how antigen presenting cells (APCs) process and present various viral antigens. Various structural and non-structural HCV proteins derived from the open reading frame (ORF) have been implicated in modulation of dendritic cells (DCs) and APCs. Besides the major ORF proteins, the HCV core region also encodes an alternate reading frame protein (ARFP or F), whose function in viral pathogenesis is not clear. In the current studies, we sought to determine the role of HCV-derived ARFP in modulating dendritic cells and stimulation of T cell responses. Recombinant adenovirus vectors containing F or core protein derived from HCV (genotype 1a) were prepared and used to endogenously express these proteins in dendritic cells. We made an intriguing observation that endogenous expression of F protein in human DCs leads to contrasting effects on activation and apoptosis of DCs, allowing activated DCs to efficiently internalize apoptotic DCs. These in turn result in efficient ability of DCs to process and present antigen and to prime and stimulate F protein derived peptide-specific T cells from HCV-naive individuals. Taken together, our findings suggest important aspects of F protein in modulating DC function and stimulating T cell responses in humans.

## Introduction

Hepatitis C virus (HCV) was first identified in 1989 as the major causative agent of parenterally transmitted and community-acquired non-A, non-B hepatitis [1]. Currently, an estimated 170 million people worldwide are chronically infected with this virus [2]. HCV is a major cause of end-stage liver diseases and a high proportion of chronic HCV carriers develop liver cirrhosis and hepatocellular carcinoma [3]. Seven major genotypes (genotype 1 to genotype 7) of HCV have been described (based on phylogenetic analyses of the core, E1, and NS5 regions of the HCV genome), with further division of each genotype into several subtypes (1a, 1b, 2c, etc.) [4], [5]. HCV contains a single stranded, positive-sense RNA genome 9.6 kb in size. This genome encodes a single open reading frame (ORF) polyprotein. This polyprotein is processed by host and viral proteases into structural (core, E1, and E2) and non-structural (p7, NS2, NS3, NS4A, NS4B, NS5A, and NS5B) proteins [6]. Apart from these ORF proteins, another protein called alternate reading frame protein (F protein) is translated from within the core encoding region by ribosomal frame shifting. During translation, a +1 ribosomal frame shift occurs at codons 9 to 11 to generate F protein with the first 10 amino acids derived from the core [7,11 and 12]. The exact role of F protein in HCV infection is not known but it is suggested that F protein is not required for HCV infection and replication [8]. However, its role in virus propagation and development of chronic disease has not been ruled out. Antibodies and cytotoxic T cells specific for the F protein have been detected in HCV infected patients, suggesting its presence during HCV pathogenesis [9]–[12].

Dendritic cells (DCs) play a critical role in initiating effective antiviral T-cell responses because DCs are one of the most potent antigen presenting cells *in vivo* [13] and play crucial roles in the enhancement or regulation of cell-mediated immune responses. All DCs express high levels of different Toll-like receptors (TLRs), an important family of pathogen recognition receptors. Ligation of these receptors results in DC maturation and inflammatory cytokine secretion, a key event in the initiation of the innate and adaptive immune response [14]. Because DCs strongly express various costimulatory and/or adhesion molecules [13], they can activate naïve T cells in a primary response.

During HCV infection, the initial interaction between virus or virus-derived proteins and DCs may contribute to either effective cellular immunity resulting in viral clearance or impaired T cell responses leading to viral persistence [15]. It has been hypothesized that viral impairment of DCs leads to failure of HCV-infected individuals to mount an effective T-cell response, and thus to develop chronic HCV infection [16]. Several studies have been reported with contradictory experimental evidence, and there is little overall consensus [17]–[30]. The differing results could be attributed to the patient population selected, the HCV viral load, the duration of infection, the cell type being studied, the *in vitro* expansion, the assay being used, etc. [15]. Further, it is not clear if DCs become impaired in chronic HCV infection, if DC impairment is a prelude to inefficient priming and maintenance of HCV-specific T cells facilitating the establishment of a chronic carrier state, or if DC impairment is a consequence of persistent and active HCV infection and associated disease progression [15]. Therefore, identifying mechanisms which lead to modulation in DC function and subsequent antigen specific T cell stimulation in HCV infection are important to understand the immunobiology of the HCV life cycle and to investigate immunotherapeutic approaches. The roles of a number of HCV ORF proteins in modulating human DCs have been extensively studied [15], [31]–[33]. The core antigen of HCV has been found to be associated with a number of immunomodulatory properties [34]–[37]. It has been suggested that most of the core gene products are contaminated with F protein due to the inherent F protein sequence in the HCV core region [38]; therefore, the effects ascribed to core proteins can be attributed to F protein or to a combination of F and core proteins. However, it has also been suggested that the production of F protein is negatively regulated by expression of the HCV core protein [38], implying that in an experimental system with abundant expression of core protein, F protein may not be sufficiently expressed. In earlier studies, we investigated the effects of HCV core protein on human dendritic cells, both as exogenously added recombinant protein and as endogenously expressed protein via recombinant adenovirus [15], [36].

In the present study we examined the role of HCV core and F proteins in modulating human DC functions and in the resultant antigen specific T cell priming. Recombinant adenoviral vectors containing HCV F or core protein were used to infect DCs obtained from HCV-naive human donors’ peripheral blood-derived monocytes. The expression of HCV F and core proteins in DCs led to upregulation of CD-95 and CD-95L, with F protein leading to significantly higher expression of both CD-95 and CD-95L and greater apoptosis of DCs. Interestingly, the endogenous expression of both core and F proteins also significantly upregulated the expression of CD-40 and TLR-3 on DC surfaces. Our results therefore provided contrasting effects of F and core proteins on DCs. In the presence of HCV F and core proteins, DCs became activated by expressing CD-40 and TLR-3 while undergoing apoptotic death due to upregulation of CD-95/CD-95L. In the next experiments, we demonstrated that apoptotic DCs were phagocytosed by live DCs; which would result in higher efficiency of DCs to prime and stimulate T cells. This theory was corroborated when these DCs were cocultured with autologous T cells; significant antigen specific priming and peptide-dependent proliferation of CD4^+^ T cells were observed. Therefore, HCV-derived core and F proteins provide a unique mechanism of DC modulation and apoptosis, ensuing in eventual T cell activation. Our results provide direct experimental evidence to coalesce two apparently contrasting observations in chronic HCV infection: DC modulation/apoptosis and induction of antigen specific T cells.

## Materials and Methods

### Cell Line and Culture

Monolayers of the 293A cell line (QBiogene Inc., CA, USA), an adenovirus-transformed human embryonic cell line that provides phenotypic complementation of E1 genes, was used for recombinant adenovirus plaque assays, amplification, and virus titration [15], [35]. A human monocytic THP-1 cell line was obtained from ATCC (Manassas, VA).

### Plasmid Construction

The F gene (amino acids 11–164) of the HCV-1 strain (genotype 1a) was PCR amplified from full-length clones of HCV using a forward primer 5′-GAA GAT CTA TGC CAA ACG TAA CAC CAA CCG TC-3′ and reverse primer 5′- GAA GAT CTC ACG CCG TCT TCC AGA ACC CGG A-3′. The PCR products were cloned into the commercial pCR 2.1 vector (Invitrogen, Carlsbad, CA, USA) and the purified cDNA fragments were cloned into the AdenoVator Transfer vector (pAdenoVator-CMV5-IRES-GFP, QBiogene) generating rAd-F. The adenovirus construct expressing HCV core protein has been reported earlier [15, 35, and 36].

### Recombinant Adenovirus Vectors

Recombinant adenoviruses were propagated, purified, and stored using the standard method provided in the manual (QBiogene) and as reported by us [35], [39]. The recombinant adenoviral vectors were stored in aliquots at −80°C. Viral particles of Ad5/CMV-LacZ (with no gene insert) obtained from QBiogene were used as a control adenoviral vector (denoted as CV throughout the manuscript).

### Preparation and Infection of Human Peripheral Blood Monocyte Derived DCs

Peripheral blood samples were obtained from HCV-negative donors (30–60 years of age of both sexes) from phlebotomy clinic after written informed consent. The HCV-negative status was based on antibody negativity. Use of human blood samples and the written consent form were approved by the Health Research Ethics Board at the University of Alberta, Canada. DCs were generated from human peripheral blood mononuclear cells (PBMCs) as described previously [35], [39]. Briefly, PBMCs were isolated from blood by Ficoll-Hypaque (Amersham Biosciences) density gradient centrifugation. The intermediate buffy layer containing PBMCs was collected and 5×10^6^ cells/ml were cultured for 2 hours in 6-well plates in RPMI 1640 medium (Invitrogen Life Technologies), supplemented with L-glutamine, 1% human AB serum (Sigma-Aldrich), 1% sodium pyruvate (Invitrogen Life Technologies), and 500 U/ml penicillin/streptomycin (Invitrogen Life Technologies). The nonadherent cells (NACs) which mostly included T cells and B cells were subsequently removed and cryopreserved to use in later assays. Remaining adherent cells were treated with 50 ng ml^−1^ granulocyte macrophage colony-stimulating factor (GM-CSF) and 10 ng ml^−1^ of IL-4 (Peprotech) in RPMI media and cultured for 6 days. On day 6, >95% of the obtained cells were double positive for CD-11c and HLA-DR, suggesting the differentiation of myeloid DCs.

### Infection with Adenovirus

DCs harvested on day 6 of the culture were infected with replication defective recombinant adenoviruses expressing HCV F or core protein, or CV at a multiplicity of infection (m.o.i.) of 100. Expression of F protein in DCs was confirmed by amplifying F protein mRNA using forward primer 5′-GAA GAT CTA TGC CAA ACG TAA CAC CAA CCG TC-3′ and reverse primer 5′- GAA GAT CTC ACG CCG TCT TCC AGA ACC CGG A-3′. HCV core protein expression was confirmed using primers reported earlier [15, 35, and 36].

### Western Blot Analysis

Western blot analyses of HCV F and core proteins were performed using reported procedures [36]. In brief, after 48 hours infection of DCs with rAd-F, rAd-core, and CV, cells were rinsed with phosphate buffered saline (PBS), lysed in 1× Laemmli buffer [50 mM Tris (pH 6.8), 2% SDS, 10% glycerol, 0.01% bromophenol blue, 100 mM DTT] and boiled for an additional 10 min. Cell lysate from each sample was loaded on a 15% polyacrylamide gel, separated by SDS-PAGE, and transferred to a nitrocellulose membrane (Bio-Rad) using a Trans-Blot apparatus (Bio-Rad). HCV-derived F or core proteins were probed with specific mAbs against F protein (gift from Dr James Ou, Department of Molecular Microbiology and Immunology, Keck School of Medicine, University of Southern California, Los Angeles, CA) or core protein (Chemicon Inc.) followed by a horseradish peroxidase-conjugated goat anti-mouse IgG antibody and enhanced chemiluminescence detection reagents (Pierce Biotechnology Inc.), as recommended by the manufacturer, to detect HCV F and core protein. β-tubulin was used as protein loading control.

### RNA Isolation, cDNA Synthesis, and Reverse Transcription

Total RNA from 2–3×10^6^ DCs was prepared using an RNA isolation kit (Roche Diagnostics, Mannheim, Germany) according to the manufacturer’s instructions, followed by cDNA synthesis from 0.5–1 µg of total RNA [36].

Phenotypic analysis of DCs infected with F and core proteins containing adenovirus vectors DCs infected with adenoviral vectors containing F or core protein were harvested after 48 hours and used to assess the phenotype by flow cytometry. The fluorescently labelled mAbs used included CD-11c (PE-Cy7), DEC-205 (FITC), HLA-DR (FITC), CD-80 (PE-Cy5), CD-86 (PE), CD-40 (PE) and DC-SIGN (APC), CD-95 FITC, CD-95L PE in different combinations (eBioscience, San Diego, CA, USA). Positive populations were selected by gating on respective isotype antibody [mouse IgG1 κ (PE-Cy7), mouse IgG2b κ (FITC), mouse IgG1 κ (PE-Cy5), mouse IgG2b κ (PE), mouse IgG1 κ (PE), rat IgG2a κ (APC), mouse IgG1 (FITC), mouse IgG1 κ (PE)] stained cells. Antibodies against various TLRs were obtained from Abcam Inc (Toronto, Canada). For flow cytometry, an intracellular staining protocol provided by the manufacturer was used for TLR-3, and TLR-8 whereas surface staining was performed for TLR-1, TLR-2, TLR-4 and TLR-5. Isotype control antibodies mouse IgG1 (FITC), mouse IgG1 (PE), mouse IgG1 κ (PE), mouse IgG1 (alexafluor 647), rabbit polyclonal IgG (PE) and mouse IgG2a (FITC) were used to gate positive population. The cells were gated on the basis of side and forward scatter and then selected for CD-11c positive cells. More than 95% of the cells were positive for CD-11c confirming the DC phenotype of the preparation (data not shown).

### Cell Purification

CD4^+^ and CD8^+^ T-cells were purified by magnetic cell sorting using magnetic beads (EasySep, Human CD4 and CD8 selection kit; StemCell Technologies, Vancouver, BC) according to the manufacturer’s instructions. Briefly, 2–3×10^7^ nonadherent cells were resuspended in 100 µl of PBS containing 2% fetal bovine serum and 1 mM EDTA and incubated with EasySep Positive Cocktail at 100 µl/ml at room temperature (RT) for 15 minutes. Magnetic nanoparticles were added at 50 µl/ml and the mixture was incubated for 10 min at RT. The volume was brought up to 2.5 ml by adding recommended buffer (PBS) containing 2% fetal bovine serum and 1 mM EDTA). The tube was placed in a magnet for 5 min. After 5 min, the supernatant fraction was poured off. This process was repeated 3 times to get pure CD4^+^ or CD8^+^ T cells. The tube was removed from the magnet and the CD4^+^ or CD8^+^ T cells that bound to the column were flushed out with 2 ml buffer (described above). Purified populations were found to be at >97% purity by flow cytometry.

### *In vitro* Proliferation Assay

Proliferative responses of T cells were measured in triplicate cultures in flat-bottom 96-well microtiter plates (Costar). A total of 2×10^5^ autologous T cells were cultured with different concentrations (10^3^ to 2×10^4^) of infected or noninfected DCs in 200 ul of AIM-V medium (Invitrogen Life Technologies) at 37°C for 5 days. Purified CD4^+^ or CD8^+^ T cells were used in these assays. Cell viability was >80% in all groups including purified CD4^+^ and CD8^+^ T cells in the beginning of the cultures. The assay included negative (no Ag) and positive (phytohemagglutin, 1 µg/ml) controls. The cells were pulsed with 0.5 µCi/well [^3^H] thymidine (Amersham Biosciences) for 16 h and harvested on nylon fibre filter papers (PerkinElmer). The levels of [^3^H] thymidine incorporation into the cellular DNA were counted in a liquid scintillation counter (MicroBeta Trilux; PerkinElmer). Tests were run in replicates of three to five wells.

To determine the secondary T cell responses against peptides derived from HCV F or core protein, replica plating assays were performed [35]. Initially, 48 wells of 96-well plates were plated with F or core protein containing adenovirus-infected DCs (10^4^/well) together with 2×10^5^ autologous purified CD4^+^ T cells in a total 200 µl/well of AIM-V medium for 5 days. On day 5, each well was split into three equal wells on three different 96-well plates. On the first plate, no Ag (antigen) was added; on the second plate, the peptides from F and core proteins were added at 1 µg/ml. On the third plate, peptides from F and core proteins were added at 10 µg/ml. Control Ags, SOD, was added in five to six replicates. Each well was fed with irradiated autologous PBMCs (1×10^5^/well) and cultured for another 5 days. At the end of the 5 days, 0.5 µCi/well [^3^H] thymidine was added, followed by harvesting the cells on day 6 and counting [^3^H] thymidine levels incorporated in the DNA.

### Detection of Apoptosis in DCs Induced by CD-95 - CD-95L Interactions

Apoptotic cells were detected by their ability to stain with FITC-conjugated Annexin V and 7-AAD (eBioscience, San Diego, CA, USA). Briefly, 5×10^5^ dendritic cells were infected with 100 m.o.i. of replication deficient recombinant adenovirus containing HCV F or core protein. Infected cells were cultured for 48 hours. At this time, cells were collected and washed 2 times with PBS and resuspended in 100 µl of 1× Annexin V binding buffer. The cells were then stained with 5 µl of FITC-conjugated Annexin V for 15 min at room temperature in the dark. The cells were washed twice with PBS and diluted with 400 µl of 1× binding buffer. Finally, 5 µl of 7-AAD was added and the cells were analyzed by flow cytometry using a FACSCanto (BD Biosciences). The stained cells were analyzed within 4 hours of staining. In the blocking experiments, anti-CD-95L antibody and isotype control mouse IgG1 (1 µg/ml) was added at the beginning of the culture.

### Expression of F or Core Protein Containing Adenovector in THP-1 Cells

F or core containing replication deficient recombinant adenovector was used to infect THP-1 cells for 48 hours and analyzed for CD-95L expression and apoptosis by flow cytometry.

### Uptake of Apoptotic DCs by live DCs

Live DCs were obtained from a 6-day culture of adherent PBMCs with IL-4 and GM-CSF as described earlier. F and control-adenovector infected DCs were obtained 24 hr after infection with recombinant adenovirus vectors. Infected DCs were stained with CFSE (5-(and 6)-carboxyfluorescein diacetate succinimidyl ester, or CFDA SE, Invitrogen, Carlsbad, USA) at 2 µM/ml concentration using the manufacturer’s instructions. For positive control of apoptotic cells, untreated DCs were cultured on a six-well dish and irradiated for 2 min with a UV transilluminator with a peak intensity of 9000 mW/cm^2^ at the filter surface and a peak emission of 313 nm [66]. Induction of apoptosis in UV treated DCs was confirmed by staining cells with Annexin V and 7-AAD (eBioscience, San Diego, CA, USA). Live DCs were cocultured at 37°C or 4°C with F protein or control vector induced CFSE labelled DCs at 1∶1 ratio. After 2 hours, flow cytometry analysis was performed to assess the uptake of CFSE-labelled apoptotic DC (CFSE^+^CD-11c^−^) by live DCs (CFSE^-^CD-11c^+^).

### Statistical Analyses

Statistical analysis was performed by one way ANOVA and t-test using Graphpad Prism (Graphpad Software Inc., La Jolla, CA, USA). *, **, and ***indicate significant differences at P<0.05, P<0.0036, and P<0.0001, respectively.

## Results

### Endogenous Expression of HCV-derived F or Core Protein in Human PBMC-derived DCs

**Figure 1:**
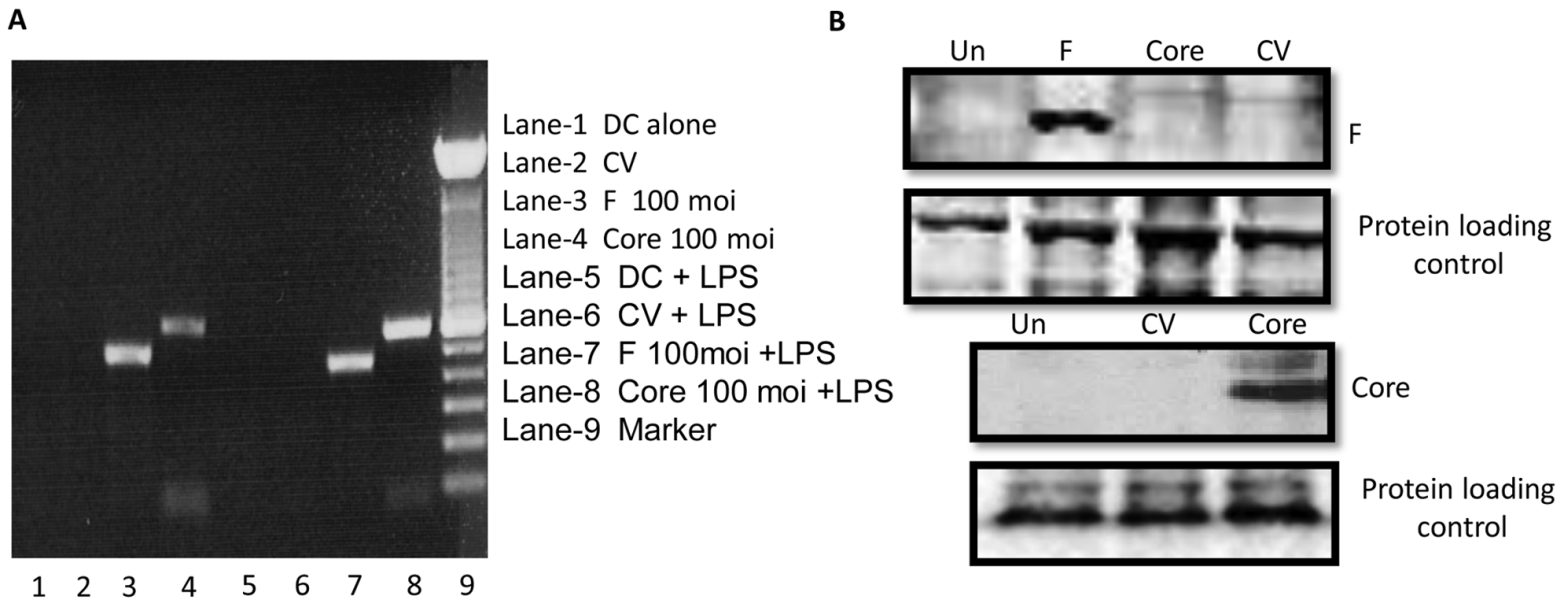
Expression of HCV F or core antigens in DCs infected with recombinant adenovirus containing HCV F or core. Panel A. **mRNA expression by RT-PCR.** DCs were infected with rAd-F or rAd-core at an m.o.i. of 100. After 48 hours of infection, DCs were harvested, mRNA was extracted, and cDNA was produced as described in Materials and Methods followed by amplification of F or core specific genes. F and core specific genes were expressed in both immature (lanes1–4) and LPS-matured DCs (lanes 5–8). Controls included DCs alone (lanes 1 and 5), and CV treated DCs (lanes 2 and 6). Panel B. **Protein expression by Western blot.** DCs were infected with rAd-F (lane 2) at m.o.i. 100 or left uninfected (lane 1); a control was infected with rAd (lane 4). 293A cells infected with rAd-core at an m.o.i. of 100 were used as negative (lane 3 of top panel) and positive control (lane 3 of the third panel), respectively. Cells were harvested 48 hours after infection. Expression of the F protein (17 kDa) was monitored by using a specific mAb against F, which precipitated a band of around 17 kDa from rAd-F infected DCs, corresponding to the putative molecular mass of HCV F. Anti-core antibody precipitated a band of ∼21 KDa from rAd-core infected DCs (third panel). β-tubulin was used as protein loading control.

Human monocyte-derived immature DCs were incubated with recombinant adenovirus vectors encoding HCV-derived F or core protein or the control (Ad/LacZ) at 100 m.o.i. for 48 hours. HCV gene expression in the DCs was determined by mRNA detection (RT-PCR) and western blotting (Figs. 1A and 1B). The F specific primer did not amplify an mRNA signal in core expressing DCs and vice versa, demonstrating the specificity of antigen expression (data not shown). The molecular masses of HCV F and core proteins were 17 and 21 kDa, respectively, corresponding to their putative molecular masses. In two repeated western blot experiments, we observed consistent protein expression at 100 m.o.i. of adenoviral vector. The cells were also observed visually under a fluorescence microscope to determine the efficiency of gene expression as represented by green fluorescent protein (GFP) expression; almost 100% of the cells were dimly positive for GFP (data not shown).

### Phenotype Analysis of DCs Expressing HCV-derived F or Core Protein

**Figure 2:**
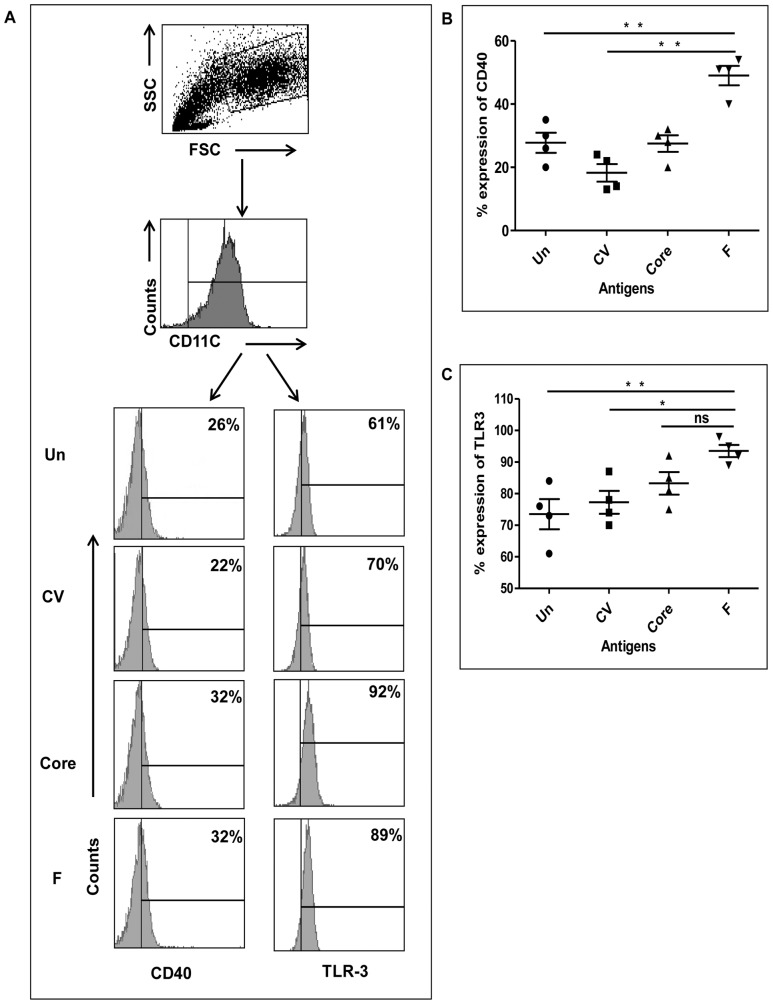
Expression of CD-40 and TLR-3 on DCs expressing F or core protein. DCs were infected with adenovirus containing HCV-derived F or core antigen. After 48 hours of infection, DCs were harvested and stained with antibodies against CD-40 (A, left panel, and B) and TLR-3 (A, right panel, and C). Data shown in panel A are representative of 4 repeated experiments and panels B and C show statistical data from four cumulative experiments.

**Figure 3:**
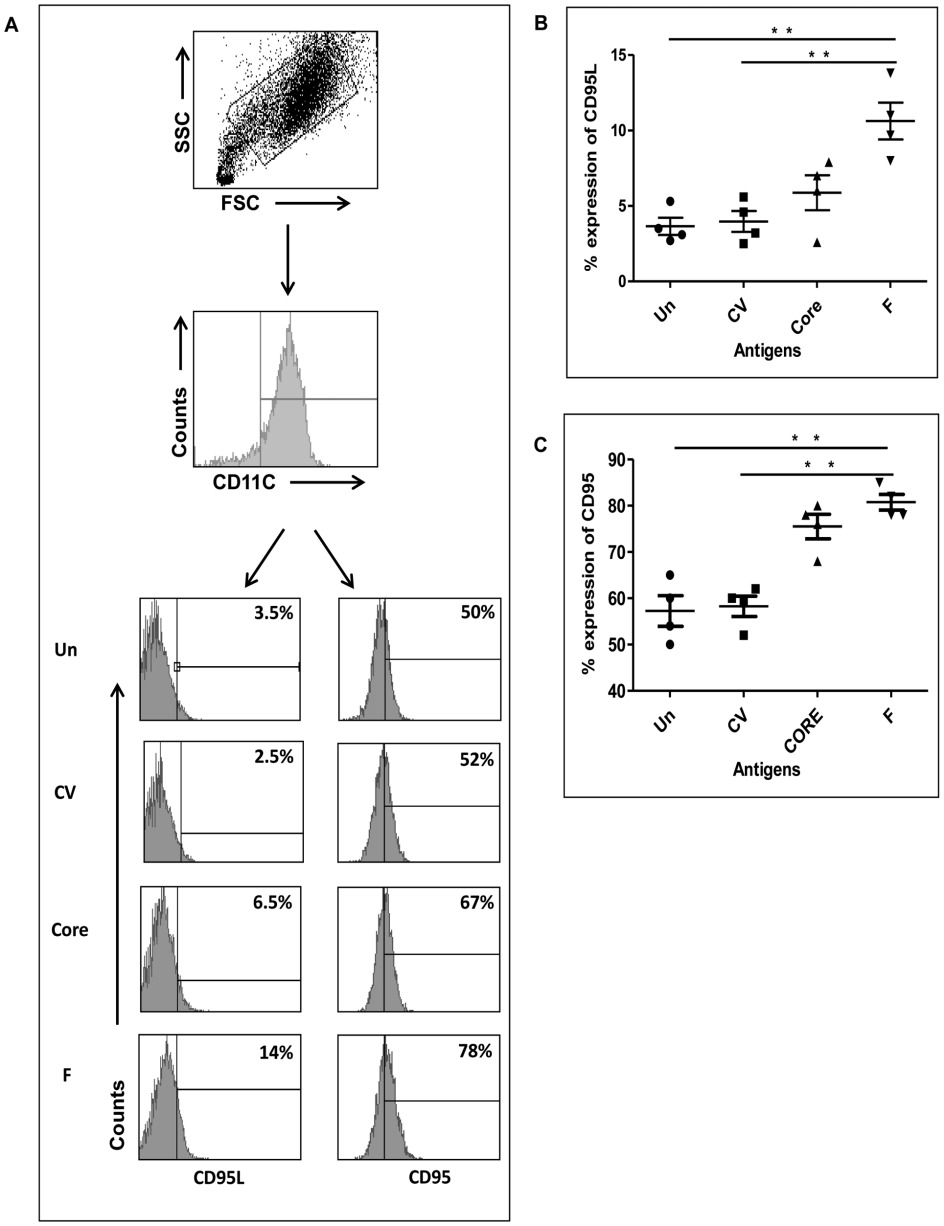
Expression of CD-95 and CD-95L in DCs expressing F or core protein. DCs were infected with adenovirus containing HCV-derived F and core antigens. After 48 hours of infection, DCs were harvested and stained with antibodies against CD-95L (A, left panel) and CD-95 (A, right panel). Data shown in panel A are representative of 5 different experiments done separately in 5 different donors. Cumulative statistical analysis of CD-95L (B) and CD-95 (C) expression is shown from four different donors.

We determined the expression of various maturation and costimulation markers such as CD-80, HLA-ABC, HLA-DR, CD-86, Dec-205, DC-SIGN and CD-40 on DCs to investigate whether the expression of HCV-derived F or core antigen led to modulation in the DC phenotype which could affect T-cell stimulation and DC survival. In addition, we analysed the expression of TLR-1, TLR-2, TLR-3, TLR-4, TLR-5, and TLR-8. In the differentiated DC cultures, CD-11c was expressed on >96% of uninfected DCs indicating the myeloid DC lineage of these cells. Upon infection with control vector or F or core recombinant adenoviral vector, CD-11c was not altered (data not shown). Also, we observed that activation/costimulation markers CD-80, HLA-ABC, HLA-DR, CD-86, Dec-205, and DC-SIGN were unchanged in DCs expressing core or F protein compared to controls (data not shown). However, we found significant upregulation in CD-40 expression (Fig. 2A, left panel, and Fig. 2B). Among TLRs, only TLR-3 was upregulated in DCs infected with HCV-derived F and core proteins (Fig. 2A, right panel, and Fig. 2C). During these experiments, we observed that in F expressing DC cultures, cellular recovery was lower (∼50%) than in core or control vector groups (data not shown). However, there was no visual sign of cell death due to toxicity in the cultures. Therefore, we examined the expression of molecules involved in apoptosis, i.e., CD-95 and CD-95L. We found that both CD-95 (Fig. 3A, right panel, and Fig. 3C) and CD-95L (Fig. 3A, left panel, and Fig. 3B) expressions were significantly higher in DCs expressing HCV-derived F protein. Expression of HCV core protein in DCs led to an increase in CD-95 expression, but not to a significant increase in CD-95L expression (Fig. 3B and 3C). The culture incubation time for the phenotype analysis was the same as in the immunofluorescence experiments and western blotting; therefore, at the time of phenotype analysis, the HCV proteins were being expressed by almost all of the DCs.

### Expression of F Protein Leads to Apoptosis in DCs

**Figure 4:**
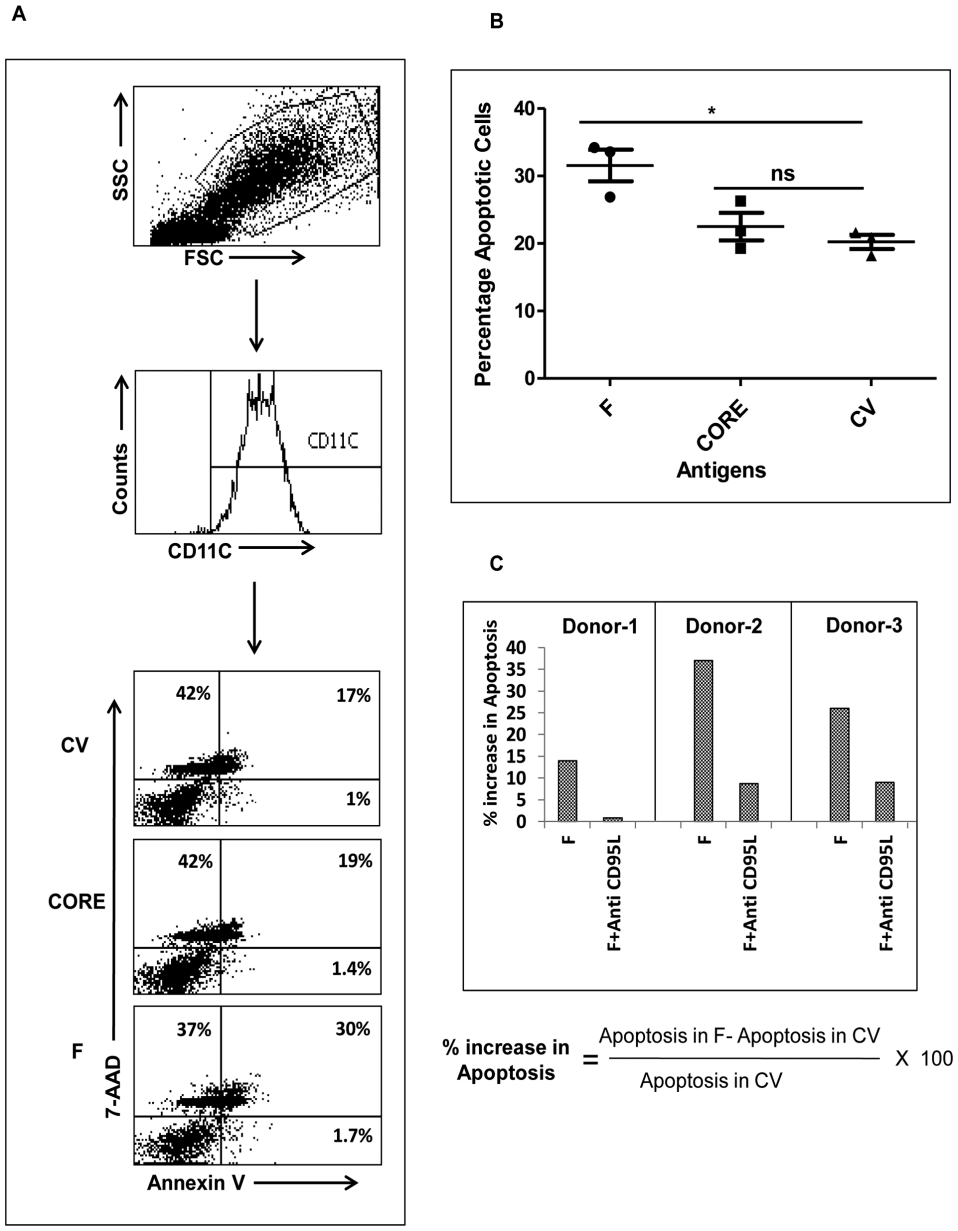
Expression of F protein leads to CD95L mediated apoptosis in DCs. DCs were infected with rAd-F, rAd-core, or control vector at an m.o.i. of 100. After 48 hours of infection, DCs were collected and stained with PE-Annexin V and 7-AAD (A). Data are representative of 3 different experiments with different donors. Statistical analysis of apoptosis in 3 different donors is shown in B. To block apoptosis, DCs were infected with rAd-F at an m.o.i. of 100 with or without 1 µg/ml of a neutralizing anti-CD-95L monoclonal antibody or isotype control antibody. After 48 hours, the cells were stained with PE-Annexin V and 7-AAD and analyzed by flow cytometry (C). The data are represented as percentage increase in apoptosis using the formula 100× (apoptosis in F - apoptosis in CV/apoptosis in CV). The experiment was repeated with three different donors and data are shown for individual donors.

We wanted to determine whether upregulation in CD-95/CD-95L expression caused apoptosis in monocyte derived dendritic cells expressing F protein. DCs infected with recombinant adenovirus containing HCV-derived F or core protein, or control vector for 48 hours were stained with PE Annexin V and 7-AAD. As shown in Fig. 4A and 4B, apoptosis was significantly higher in DCs infected with HCV-derived F expressing adenovirus compared to control vector and core groups. A representative flow cytometry analysis is shown in Fig. 4A and statistical analysis from three different experiments is shown in Fig. 4B. To confirm that apoptosis is mediated by CD-95L interaction with CD-95, neutralizing anti-CD-95L antibody was added to the culture at the time of initial infection with adenovirus (Fig. 4C). Addition of neutralizing anti-CD-95L antibody [40] at 1 µg/ml significantly reduced the apoptosis in DCs infected with HCV-derived F expressing adenovirus compared to the isotype control antibody-treated group (Fig. 4C).

### Expression of F Protein in THP-1 Cells Induces Upregulation in CD-95L Expression Followed by Apoptosis

**Figure 5:**
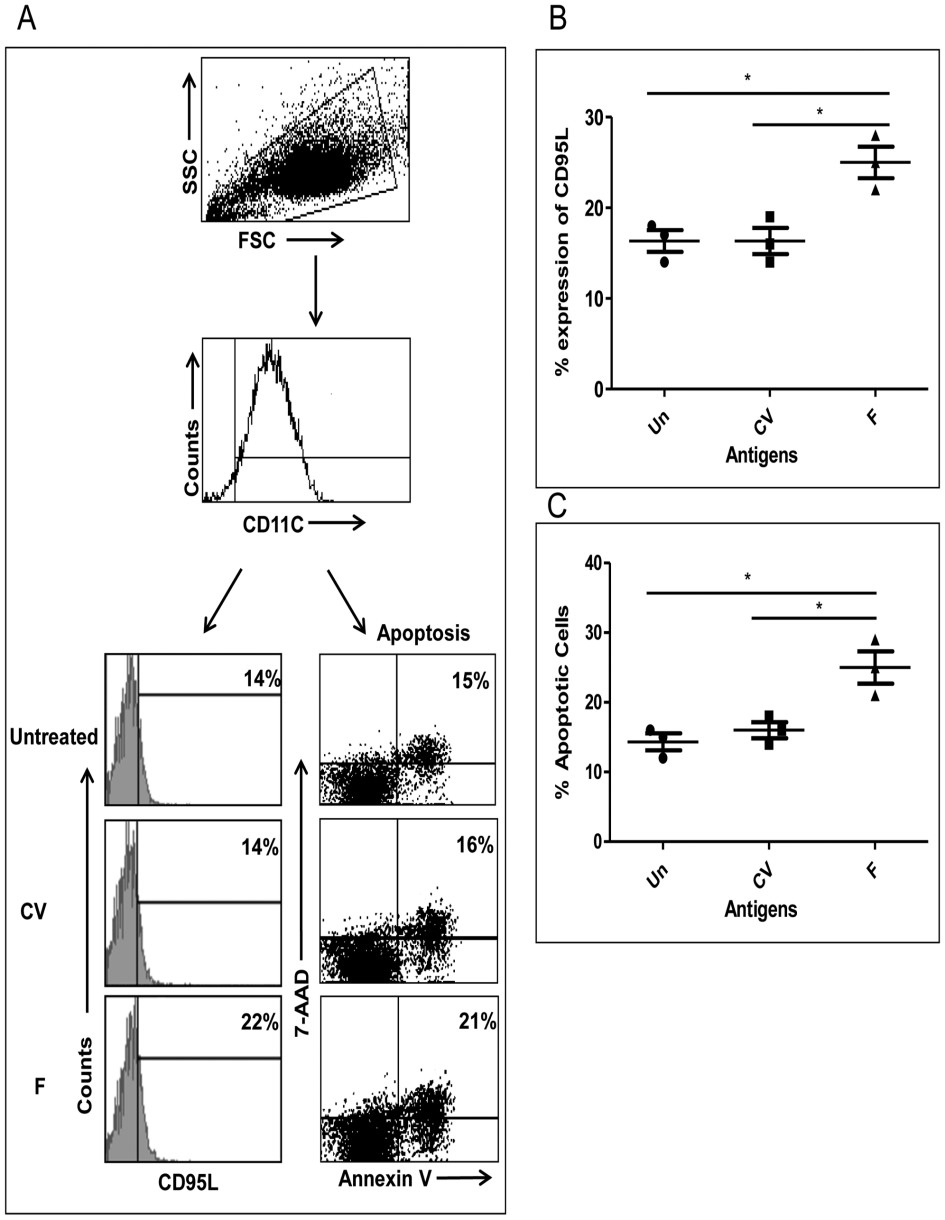
rAd-F infection of THP-1 cells induces CD-95L expression and apoptosis. THP-1 cells were infected with rAd-F at an m.o.i. of 100. After 48 hours of infection, the cells were harvested and stained using an anti-human CD-95L PE-conjugated monoclonal antibody and analyzed by flow cytometry (A left panel and B). To measure apoptosis in THP-1 cells, after 48 hours of rAd-F infection, THP-1 cells were harvested, stained with PE-Annexin V and 7-AAD, and examined by flow cytometry (A right panel and C).

After observing the effects on CD-95L expression and apoptosis in primary monocyte derived human DCs, we determined if similar effects of HCV-derived F protein could be seen in the human monocytic cell line THP-1. The rationale was that in primary monocyte differentiated DC cultures, contaminating cells could provide accessory functions to induce CD-95L and apoptosis, whereas in the THP-1 cell line we would be looking at a direct effect of F protein expression on CD-95L and apoptosis. THP-1 cells were infected with adenovirus vectors containing F or control vector and examined for CD-95L expression after 48 hours by flow cytometry (Fig. 5). The infection with adenovirus expressing F protein led to an increase in CD-95L expression on the surface of the infected THP-1 cells, whereas the control vector did not influence CD-95L expression relative to untreated THP-1 cells (Fig. 5A, left panel and Fig. 5B). In accordance with the CD-95L expression, apoptosis, as measured by Annexin V and 7-AAD staining, was enhanced in THP-1 cells infected with adenovirus vector expressing F protein, compared to control vector or untreated cells (Fig. 5A, right panel and Fig. 5C).

### Uptake of Apoptotic DCs by Live DCs

**Figure 6:**
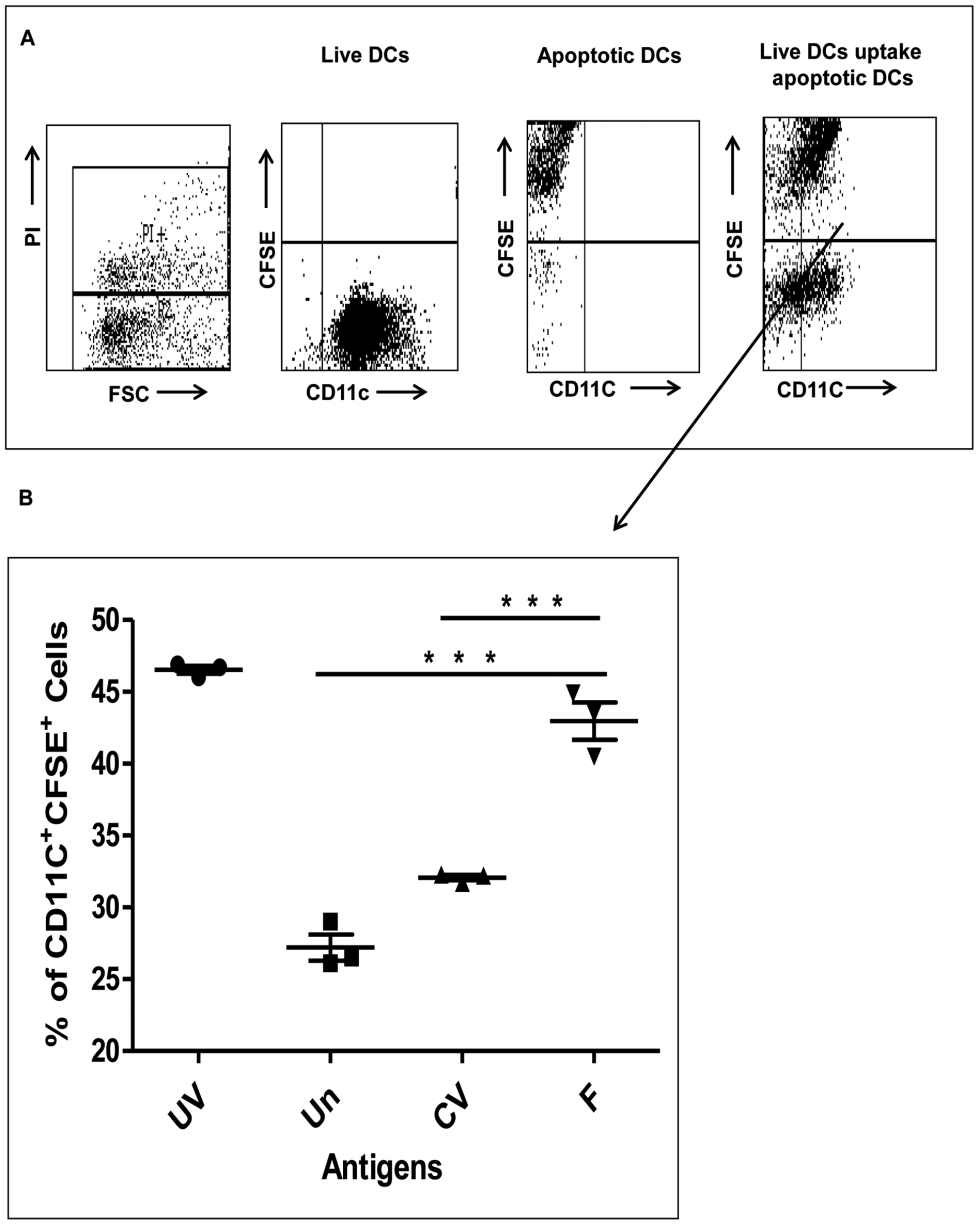
F-protein induced apoptotic DCs are efficiently taken up by live DCs *in vitro*. CFSE-labelled apoptotic DCs were incubated with live immature DCs at a ratio of 1∶1 for 2 hours. Flow cytometry analyses were conducted to assess uptake of CFSE^+^ apoptotic DCs by live CD-11c^+^ DCs. Double positive cells indicate the number of live DCs that have uptaken apoptotic DCs. (A) the gating pattern in different groups: live DCs were gated based on PI exclusion and CD-11c expression and the proportion of CFSE^+^ cells was assessed among CD-11c^+^ to determine the phagocytosed DCs. (B) CFSE^+^CD-11c^+^ double positive cells are significantly higher in the F group which has more apoptotic DCs induced by rAd-F infection compared to control vector or untreated DCs. UV treated DCs were used as positive controls for apoptotic DCs. Data are representative of three different experiments.

The induction of apoptosis in DCs by HCV-derived F protein could be both beneficial and detrimental for efficient antigen presentation of HCV antigens. In our experiments (Figs. 4 and 5), however, we observed apoptosis in a limited percentage of DCs or THP-1 cells. We therefore hypothesized that induction of apoptosis in a small number of DCs might allow for better phagocytosis by live DCs, resulting in efficient antigen presentation and T cell stimulation. To assess the phagocytosis of F protein induced apoptotic DCs by live DCs, F expressing apoptotic DCs were labelled with CFSE and incubated with immature live DCs not expressing F protein. Eight hours later, flow cytometry analysis was performed to assess the uptake of CFSE-labelled apoptotic DCs by live DCs (PI^–^CD-11c^+^) (Fig. 6). The results indicated that in the F expressing group with a high percentage of apoptotic DCs, the percentage of CFSE^+^CD-11c^+^ DCs was increased compared to control vector or untreated CFSE labelled DCs (Fig. 6). The UV treated DC group used as a positive control showed the highest number of CFSE^+^CD-11c^+^ DCs. To confirm that there were no contaminating CFSE^+^ PI^–^ apoptotic DCs, a parallel experiment was performed in which apoptotic DCs were labelled with CFSE, cultured for 2 hours, and subsequently stained with propidium iodide (PI); approximately 98% of the DCs were PI^+^ (data not shown), indicating that gating for PI^–^ cells would gate out any CFSE^+^ apoptotic DCs. Furthermore, to distinguish binding of apoptotic DCs to live DCs from internalization of apoptotic DCs by live DCs, the coculture experiments were carried out at 4°C; under these conditions, phagocytosis was completely inhibited (data not shown). Collectively, the results indicated that high apoptosis induced in DCs by F protein of HCV led to increased phagocytosis by live DCs.

### T Cells from HCV-naive Donors Proliferate upon Stimulation with Autologous DCs Endogenously Expressing HCV-derived F or Core Antigen

**Figure 7:**
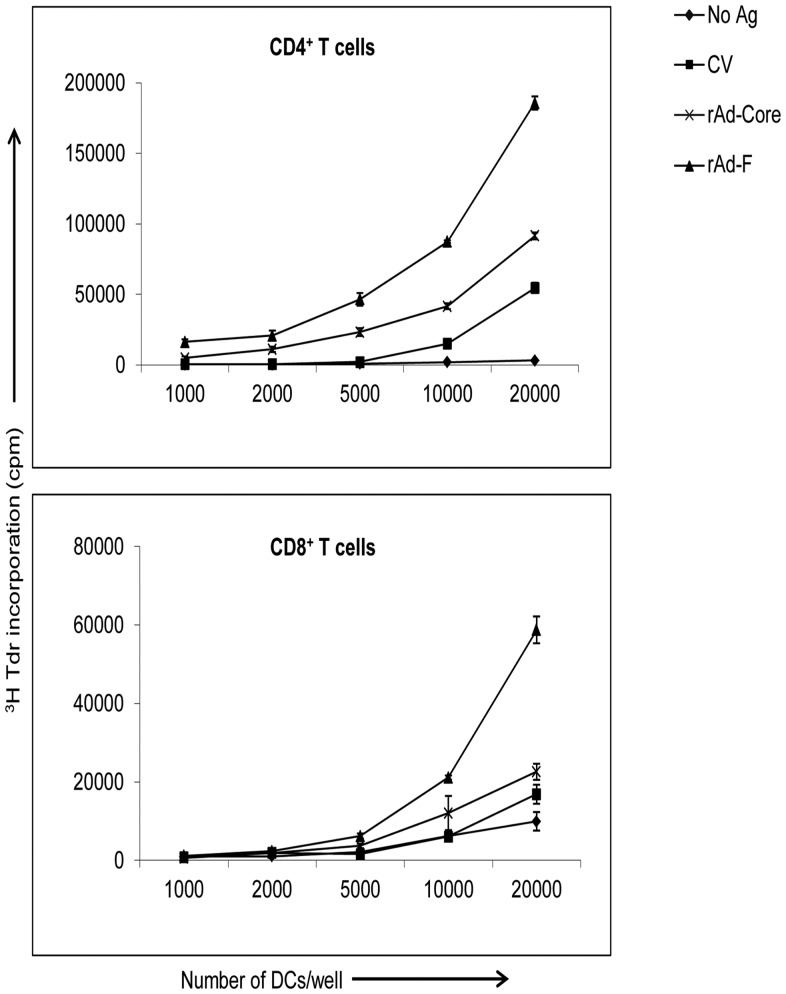
Primary proliferative response of naive autologous CD4^+^ and CD8^+^ T cells upon stimulation by DCs expressing HCV F and core antigens. Purified CD4^+^ and CD8^+^ T cells were stimulated using DCs expressing HCV antigens at various DC:T cell ratios. The proliferation of T cells was determined by a ^3^H-thymidine incorporation assay. The lines on the graphs represent DCs with no antigens, DCs expressing control vector, core protein, and F protein according to the figure legends. Mean and standard deviations of triplicate wells are shown. The data are representative of 5 repeated experiments with 5 different donors.

In earlier studies [35] we demonstrated that naive T cells from uninfected donors can be primed *in vitro* against core and NS3 antigens of HCV by using autologous DCs expressing these antigens through recombinant adenovirus vectors. Here we found that F protein leads to apoptosis in DCs but results in more phagocytosis by live DCs. We wondered if naive T cells from uninfected donors could be stimulated *in vitro* to proliferate against HCV-derived F antigen. Core antigen was used as a positive control in these experiments. The immature dendritic cells (iDCs) expressing HCV F or core antigen were cultured with autologous purified CD4^+^ or CD8^+^ T cells for 5 days at DC:T cell ratios of 1∶200 to 1∶20. Uninfected DCs or control-vector infected DCs were used as negative controls (Fig. 7). T-cell proliferation was determined as a measure of T-cell stimulation. These experiments were performed with T cells obtained from five individual donors and of these, HCV antigen-dependent T-cell proliferation responses were obtained from four donors. After 5 days of culture, *in vitro* F or core-specific proliferation was significantly higher in all the donors than in negative control groups. Proliferation against HCV-derived F or core was significantly higher than against uninfected DCs or control vector-infected DCs (P<0.05 at DC:T cell ratios of 1∶40 and 1∶20 in all four donors).

### CD4^+^ T Cells Primed *in vitro* by Autologous DCs Expressing HCV F or Core Antigen Proliferate in a Peptide-Specific Manner in Secondary Culture

**Figure 8:**
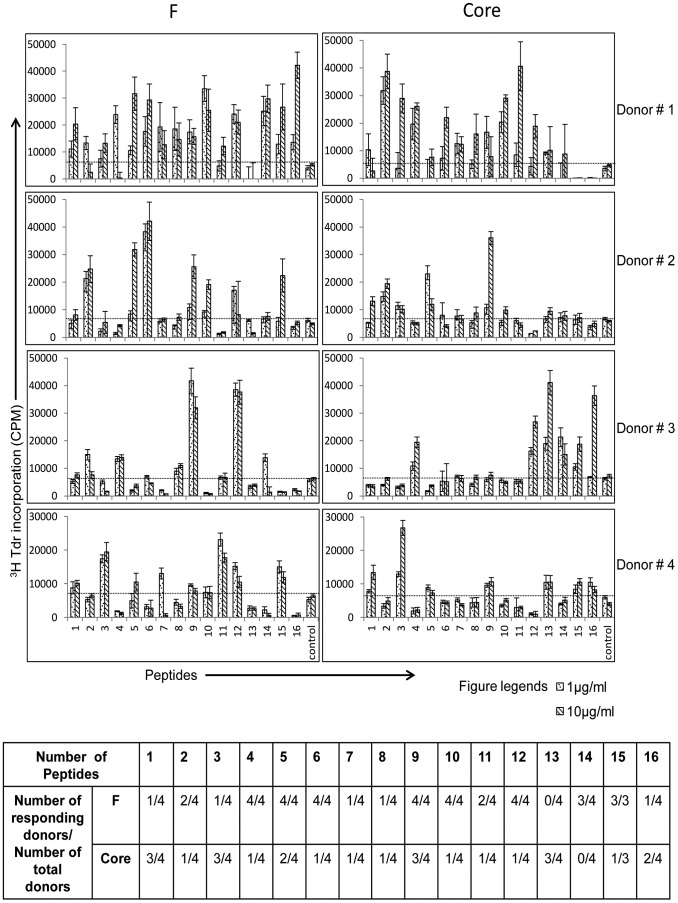
*In vitro* priming with DCs expressing F and core proteins leads to peptide dependent proliferation of CD4^+^ T cells in secondary cultures. The counts per minute (CPMs) shown are mean ± standard error of individual CPMs subtracted with no-antigen groups from corresponding wells. The data are shown from four experiments with four different donors. A response was considered positive when the CPM in the presence of peptide was more than 5,000 after subtracting with no-antigen groups, and was higher than the control peptide group. Core peptide was used as a control in F-stimulated group and vice-versa. The bottom table summarizes the peptide specific responses from all four donors.

CD4^+^ T cells were stimulated with autologous DCs expressing HCV-derived F or core containing adenovirus vector, followed by a replica-plating experiment to determine peptide-specific proliferation of CD4^+^ T cells [35]. Peptide-specific proliferation was evident in the purified CD4^+^ T cells in the secondary cultures with HCV F and core peptides at both 1 µg/ml and 10 µg/ml concentrations (peptides used are shown in Table-1). As controls, we also stimulated naive CD4^+^ T cells in primary cultures with DCs infected with HCV-derived F and core protein but in secondary culture no peptides were added. In addition, F derived peptides were used as a negative specificity control antigen in core-stimulated cultures and vice versa. CD4^+^ T cell proliferation from the no peptide group was subtracted in peptide stimulated groups. The experiment was repeated in four different donors (Fig. 8). We calculated a cumulative response score against F and core derived peptides in all four donors using the following formula: sum of total number of responders for individual peptides/sum of total number of peptides tested from all donors. The results were 39/64 for F peptides and 25/64 for core peptides, suggesting that F antigen is either more immunogenic than core antigen or it is able to efficiently stimulate T cells.

**Table 1:** Peptide sequences from core and F protein.

No.	Position	AA Sequence
A. Peptides derived from F protein		
1	01–15	MSTNPKPQRKPNVTP
2	11–25	PNVTPTVAHRTSSSR
3	21–35	TSSSRVAVRSLVEFT
4	31–45	LVEFTCCRAGALDWV
5	41–55	ALDWVCARRGRLPSG
6	51–65	RLPSGRNLEVDVSLS
7	61–75	DVSLSPRHVGPRAGP
8	71–85	PRAGPGLSPGTLGPS
9	81–95	TLGPSMAMRVAGGRD
10	91–105	AGGRDGSCLPVALGL
11	101–115	VALGLAGAPQTPGVG
12	111–125	TPGVGRAIWVRSSIP
13	121–135	RSSIPLRAASPTSWG
14	131–145	PTSWGTYRSSAPLLE
15	141–155	APLLEALPGPWRMAS
16	148–162	LPGPWRMASGFWKTA
B. Peptides derived from Core protein		
1	1–15	MSTNPKPQRKTKRNT
2	13–27	RNTNRRPQDVKFPGG
3	25–39	PGGGQIVGGVYLLPR
4	37–51	LPRRGPRLGVRATRK
5	49–63	TRKTSERSQPRGRRQ
6	61–75	RRQPIPKARRPEGRT
7	73–87	GRTWAQPGYPWPLYG
8	85–99	LYGNEGCGWAGWLLS
9	97–111	LLSPRGSRPSWGPTD
10	109–123	PTDPRRRSRNLGKVI
11	121–135	KVIDTLTCGFADLMG
12	133–147	LMGYIPLVGAPLGGA
13	145–159	GGAARALAHGVRVLE
14	157–171	VLEDGVNYATGNLPG
15	169–183	LPGCSFSIFLLALLS
16	177–191	FLLALLSCLTVPASA

## Discussion

Hepatitis C is a major blood-borne disease, causing chronic infection in 65–80% of infected patients. The hepatitis C virus (HCV) has been shown to infect several extrahepatic cells including dendritic cells (DCs) [41]–[44]. DCs are the major antigen presenting cells; they take up a variety of antigens and process and present them to CD8^+^ and CD4^+^ T cells in the context of MHC molecules. After clonal expansion the activated T cells become effector cells and mount an antigen-specific immune response to clear the infection. It has been proposed that HCV persists in a majority of infected individuals due to lack of efficient adaptive immune responses [45]–[47]. It has been hypothesized that HCV can modulate DC and/or T-cell function, and DC dysfunction is known to be one of the direct mechanisms that enable viral persistence [20]. Earlier work by Bain et al. and Kanto et al. [17], [19] provided evidence for DC dysfunction in chronic HCV infection. Several studies show impairment of DC function in HCV-infected individuals [18,20,25, and 26]; however, other reports suggest that DC functions are not affected [21]–[23], [27]–[29]. In chronic hepatitis C, factors leading to DC dysfunction are postulated to be first, a direct infection of DCs and second, the presence of HCV proteins that might modulate DC function [37], [48]. The evidence for direct infection and replication of HCV in DCs still remain elusive, and it can be envisioned that DCs can acquire HCV-derived proteins through phagocytosis of infected hepatocytes, allowing the apparent expression of HCV proteins in DCs. However, under this scenario, the levels of expression of various HCV proteins would be substantially low albeit for prolonged period of time due to chronic nature of HCV infection. It is also possible that upon uptake of HCV RNA within the DCs, the translation of the viral RNA allows for substantial HCV-protein levels, even in the absence of viral replication within the DCs.

In this paper we show for the first time that HCV-derived F protein can modulate DC functions and the overall immune response against HCV. F protein is synthesized due to a ribosomal frame shift at codon 11 in the HCV core encoding region. It was suggested that F protein is not required for infection and replication of HCV [8] but its role in viral persistence and development of chronic hepatitis and hepatocellular carcinoma has not been ruled out.

We used recombinant adenovirus vectors containing HCV F or core protein to endogenously express these antigens in DCs. Infection with recombinant adenovirus vectors led to expression of HCV F or core protein, as indicated by immunofluorescence in ∼100% of the DCs [49]. Further, mRNA detection by RT-PCR (Fig. 1A) and protein detection by western blotting (Fig. 1B) confirmed the expression of antigens in DCs. These initial experiments verified our ability to efficiently express HCV F and core proteins in human DCs.

Upon examining the phenotype of DCs expressing HCV F, core protein, or control vector, we did not observe significant differences in the expression of CD-11c, CD-80, CD-86, DEC-205, DC-SIGN, HLA class I, or MHC class II (data not shown). However, we found significant upregulation in CD-40 (Fig. 2A, left panel, and Fig. 2B) in DCs expressing F protein.

Because there was significantly higher expression of CD-40 on F-expressing DCs, we hypothesized that upregulation of CD-40 alone may not be sufficient for efficient stimulation of T cells. Therefore, we decided to look for other markers that might be involved in stimulation of T cells. From previous reports [50], [51] we noted that CD-40 and Toll-like receptors (TLRs) together can efficiently stimulate T cells. It has been reported that in situ stimulation of CD-40 and TLR-3 transforms ovarian cancer-infiltrating DCs from immunosuppressive to immunostimulatory cells [50]. It has also been shown that combinatorial stimulation of TLRs and TRAF signalling by CD-40 cross–linking generates a 10–20 fold increase in the number of activated CD8^+^ T cells compared to either agonist alone [51]. Synergistic effects of combined CD-40 and TLR-3 agonists produced one of the best T cell responses in healthy mice [51]. In murine nonepithelial tumours, CD-40/TLR-7 agonists have been utilized as adjuvants in vaccination with exogenous tumour antigen, which resulted in stronger and less toxic antitumour memory T-cell responses compared to monotherapy [52]. CD-40 signaling synergizes with TLR-2 in the BCR independent activation of resting B cells [53]. Therefore, we examined the expression of various TLRs: TLR-1, TLR-2, TLR-3, TLR-4, TLR-5 and TLR-8 on HCV F or core expressing DCs. Interestingly, we observed a significant increase in intracellular TLR-3 expression in F expressing DCs but not in core expressing DCs compared to control vector expressing DCs or untreated DCs (Fig. 2A, right panel). The remaining TLRs were unchanged within different experimental groups (data not shown).

In contrast to activation molecules such as CD-40 and TLR-3, increased expression of CD-95 and CD-95L can lead to apoptosis in cells [54], [55]. It has also been reported that DCs can undergo apoptosis involving several mechanisms, including CD-95/CD-95L upregulation [56], [57]. In the case of HCV, it was shown that expression of core protein induced apoptosis in response to anti-CD-95 monoclonal antibody [58] and enhanced the susceptibility of hepatocytes to TNF-**α** mediated apoptosis [59]. Moreover, CHO cells stably expressing HCV core protein were shown to undergo apoptosis in response to serum starvation [60]. The apoptotic effect of HCV NS3 protein on three main subsets of cytotoxic lymphocytes prevalent in liver tissue chronically infected with HCV has been shown [61].

Here we report that the expression of HCV-derived F protein in DCs leads to increased expression of CD-95 and CD-95L (Fig. 3). HCV core protein led to a less substantial increase in CD-95L expression compared to the control vector (Fig. 3). Further, the endogenous expression of F protein in DCs led to significantly high apoptotic death in DCs, which was detected by staining with PE-Annexin V and 7-AAD (Fig. 4A and 4B). Antibodies against CD-95L are known to block apoptosis mediated via CD-95L, so the DCs were infected with recombinant adenovector expressing F protein and cultured in the presence of anti-CD-95L antibody (Fig. 4C). We observed that the presence of anti-CD-95L blocked the apoptosis induced by HCV F protein in DCs (Fig. 4C).

To confirm our observation in primary DCs we did similar experiments in THP-1 cells, a human monocyte derived cell line. After infecting THP-1 cells with adenovirus vector containing HCV-derived F protein, we observed upregulation in CD-95L expression (Fig. 5A, left panel and Fig. 5B) and apoptosis (Fig. 5A, right panel and Fig. 5C).

In our experiments with primary DCs and the THP-1 cell line, we observed that only a fraction of DCs upregulated CD-95L/95 and underwent apoptosis despite ∼100% antigen expression in DCs. The reason for this is not clear but we speculate that DC heterogeneity may be a contributing factor. Although the *in vitro* culture led to differentiation of monocytes into DCs, there are possibly a variety of DCs with individual markers, maturation, and cytokine profiles that could affect their progression to apoptosis under the influence of F protein. At this time it is not clear whether CD-40, TLR-3, CD-95, and CD-95L are upregulated in different DC populations or in the same cells, and the mechanism behind this upregulation is also unclear.

Apoptosis plays a very important role in the generation of strong immune responses [62], [63]. Several reports demonstrate apoptosis of DCs in viral and other pathogenic infections [56,64, and 65]. Different groups have suggested different roles for apoptosis; three examples follow. Measles virus induced Fas L mediated apoptosis in DCs helps in release of the measles virus [56]. Apoptosis induced in DCs by pathogen *Legionella pneumophila* helps in restricting its intracellular replication [64]. Uptake of apoptotic DCs leads to conversion of live DCs into tolerogenic DCs; these tolerogenic DCs are unable to mature, secrete TGF-β1, and induce generation of Foxp3^+^ Tregs [66]. In contrast, it has been shown previously that human DCs can acquire relevant antigens and stimulates MHC class I-restricted cytotoxic T lymphocytes (CTLs) by phagocytosing apoptotic cells [67], [68]. Therefore, we examined the internalization of apoptotic DCs induced by F protein by live DCs (Fig. 6) [66]. The results obtained indicated that the number of CFSE^+^CD-11c^+^ cells is highest in the positive control group where maximum apoptosis was observed (37% in the UV group, Fig. 6). Similarly, untreated DCs and control vector treated DCs with low numbers of apoptotic DCs, had lower numbers of CFSE^+^CD-11c^+^ cells (7% and 15%, respectively) compared to F expressing DCs (34% in the F treated group, Fig. 6). To confirm that there were no contaminating CFSE^+^ PI^–^ apoptotic DCs, a parallel experiment was performed in which apoptotic DCs were labelled with CFSE, cultured for 2 hours, and subsequently stained with PI; approximately 98% of the DCs were PI^+^ (data not shown), indicating that gating for PI^–^ cells would gate out CFSE^+^ apoptotic DCs.

So far our results depicted an interesting scenario: endogenous expression of F protein led to substantial upregulation in CD-40/TLR-3 expression, apoptosis in DCs through CD-95/CD-95L expression, and efficient uptake of apoptotic DCs by live DCs. Therefore, we hypothesized that apoptosis induced by F protein in DCs is in fact helping in further activation of DCs and ultimately stimulated antigen specific T cells.

Therefore, we cocultured DCs expressing HCV F or core protein, control vector treated DCs or untreated DCs with autologous CD4^+^ and CD8^+^ T cells (Fig. 7). As an initial measure of T-cell activation, purified CD4^+^ and CD8^+^ T cells were used. In 4 out of 5 donors tested; we observed antigen-dependent proliferation in core and F groups, compared with control vector or uninfected DCs. Interestingly, proliferation was significantly higher in the F stimulated group compared to core stimulated group in both CD4^+^ and CD8^+^ T cells (Fig. 7). Also, the stimulation with CV-infected DCs was much lower than in DCs expressing F or core antigen, suggesting that adenoviral antigens may be much less stimulatory than HCV F or core antigen. In an earlier report we demonstrated efficient priming and stimulation of antigen specific T cells *in vitro* using autologous DCs expressing core antigen [35].

In our next experiments we sought to further confirm the antigen specific CD4^+^ T cell proliferation after initial *in vitro* priming (Fig. 8A and 8B). For these experiments, purified CD4^+^ T cells were incubated with the DCs expressing HCV F or core antigen in the priming cultures and restimulated with irradiated autologous PBMCs along with synthetic peptides derived (Table-1) from F or core protein in the secondary cultures. Since it is expected that the frequency of antigen-specific T cells would be very low in HCV-naive individuals, we performed these experiments in replica-plating cultures [35]. The proliferative responses of CD4^+^ T cells in replica-plating experiments provided conclusive evidence of antigen specific T-cell proliferation in the primary *in vitro* cultures and also of *in vitro* priming of T cells against peptides of F or core using autologous DCs expressing these HCV antigens. We calculated a cumulative response score for quantification of antigen specific responses as described in the results section and observed that for the four donors tested, much more stimulation of peptide specific T cells was observed with F protein (score = 39) than with core protein (score = 25). It is possible that the higher stimulation of peptide specific T cells is related to the higher immunogenicity of F protein compared to core protein, but the ability of F protein to significantly modulate DCs could be a contributing factor.

Based on our results we propose a model to explain how specific immune response and apoptosis can simultaneously occur after expression of HCV F protein in DCs. Endogenous expression of F protein leads to DC activation; activated DCs express CD-40 and TLR-3 and some DCs upregulate CD-95L/95; upregulation of CD-95L/95 leads to DC apoptosis; the apoptotic DCs are phagocytosed by live DCs; the increased DC activity allows better antigen processing and presentation by DCs.

It is well known that phagocytosis of apoptotic cells by DCs leads to further activation of DCs [67]. Coculturing of the activated DCs with autologous T cells results in efficient priming and stimulation of antigen specific T cells, which can be identified by measuring peptide specific proliferation. Therefore, HCV-derived core and F protein antigens provide a unique mechanism of DC modulation and apoptosis, ensuing in T cell activation. The detailed functional characteristics of stimulated T cells are currently being investigated in our laboratory.

The role of apoptosis in HCV infection is not well defined. The kinetics and the extent of hepatocyte apoptosis as well as the pro- and anti-apoptotic mechanisms involved remain unclear. It remains to be tested whether enhanced apoptosis of hepatocytes in HCV infection is related to viral clearance, and whether it has a therapeutic benefit [69]. In addition, apoptosis of DCs *in vivo* in chronic HCV infection has been reported but remains to be clearly established. However, these investigations are challenged by the compounding factors and complexity of *in vivo* situations.

Our studies reveal the potential of an HCV antigen (F or ARFP) that has not attracted much attention in most of the studies done to date. A number of studies examining immune responses have used PBMCs and T cells from chronically infected HCV patients, whose function and activation are severely modulated/modified due to the long-term presence of infection. Further, *in vivo*, all antigens of HCV are almost simultaneously present, compounding the overall effects on the immune system. Our experimental system approaches HCV through individual antigens and delineates the potential of each in terms of a unique mechanism of immune modulation and stimulation. Our results provide important steps towards understanding the mechanism of immune modulation by HCV-derived F protein.
